# Accelerometer-based and self-reported physical activity of children and adolescents from a seasonal perspective

**DOI:** 10.3389/fspor.2023.1294927

**Published:** 2024-01-03

**Authors:** Melanie Eckelt, Djenna Hutmacher, Georges Steffgen, Andreas Bund

**Affiliations:** ^1^ Department of Education and Social Work, University of Luxembourg, Esch-sur-Alzette, Luxembourg; ^2^ Department of Behavioural and Cognitive Sciences, University of Luxembourg, Esch-sur-Alzette, Luxembourg

**Keywords:** physical activity, accelerometer, self-report, children, adolescents

## Abstract

**Background:**

Many children and adolescents in Europe are insufficiently physically active, which makes the advancement of children’s physical activity a critical health promotion target. However, there are some environmental factors, such as the amount of daylight, weather conditions, temperature, and precipitation levels, which might influence physical activity behavior. The purpose of this study was to assess accelerometer-based and self-reported daily physical activity of children and adolescents in Luxembourg, during autumn/winter as well as during spring/summer, and to examine if there is a seasonal influence on the physical activity behavior.

**Methods:**

At two measurements, one in autumn/winter and one in spring/summer, physical activity of *N* = 137 (59.12% females; *M* = 12.37 years) participating children and adolescents aged 10–18 years was objectively undertaken via an accelerometer (ActiGraph) and subjectively assessed using, among others, one item of the MoMo physical activity questionnaire.

**Results:**

A repeated measures ANOVA revealed a significant seasonal effect on moderate to vigorous physical activity per day [*F*(1.000, 135.000) = 7.69, *p* < 0.05, partial *η*² = 0.054]. More minutes of moderate to vigorous physical activity per day were accrued in spring/summer than in autumn/winter. The mean difference scores between the accelerometer-based and the self-reported physical activity at the two time periods, T1 and T2, correlated significantly (*r* = 0.31, *p *< 0.001).

**Conclusions:**

According to these results, children and adolescents are less physically active in autumn/winter than in spring/summer. However, the discrepancy between the accelerometer-based and the self-reported physical activity remains stable over the two measurements. Therefore, schools, sports clubs, and communities should offer special physical activity programs for the colder season.

## Introduction

Physical activity (PA) has beneficial effects on health, such as increased physical fitness and vitality, increased self-confidence, and a reduced risk of obesity and related diseases, and therefore is highly recommended for children's and adolescents' wellbeing (1, 2). Further health benefits for children and adolescents associated with PA include cardiometabolic health, motor skill development, bone density, and emotional regulation/psychological health (3, 4). Due to its great importance for development and health, the PA of children and adolescents has become a key issue in research over the last decade. Nevertheless, a global school-based survey conducted across 146 countries by Guthold et al. (5) recently revealed that 81% of the adolescents are insufficiently physically active, and thus, do not meet the World Health Organization (WHO) guideline (2020) of being active on a moderate to vigorous level for an average of 60 min per day (6).

As the promotion of children's PA is a critical health target, it is important to develop effective interventions to increase the PA, and thus, it is inevitable to understand why, when, and how much PA is performed (7)*.* In terms of the socio-ecological model, the promotion of health should focus on intrapersonal behavioral factors as well as the interrelationships between individuals and the social, physical, and policy environment that influence the specific behavior (8). It has been suggested that a comprehensive approach, such as the socio-ecological model, is essential for examining the multilevel factors that may determine PA. The model supports the identification of opportunities to promote PA by considering the individual, behavioral, social, and physical environmental factors that may influence a person's ability to be sufficiently physically active (8, 9).

In the natural environment, changes in weather were found to have an impact on motivation for moderate to vigorous physical activity (MVPA) (10). It has been observed that children's PA levels reveal a seasonal pattern (11). PA levels are generally low in winter, when dark evenings and cool, wet weather is found to be inhibiting PA (12). Hence, it has already been shown that MVPA levels are higher during spring and summer (13). One study observed a 15%–30% lower MVPA in autumn and winter than in spring (14). Lewis et al. (15) reported that conditions for MVPA seem to be optimal when the environmental temperature ranges between 20°C and 25°C, and that rainfall is negatively associated with MVPA and positively, with sedentary time [SED time; (15)].

With knowledge of seasonal fluctuations in children's health behavior, interventions to change behavior can be designed more precisely. In this way, more interventions can be offered in the periods of the year when the activity level is lowest (14). Therefore, it is important to identify the seasons characterized by low activity levels to design targeted public health interventions that promote physical activity in children (16). However, the association of season with PA and SED time is likely to be country-specific, because weather differs greatly between countries (17). This is one reason to conduct the analysis with data of Luxembourg for the first time.

In general, PA can be assessed using a variety of methods including behavioral observations, questionnaires, PA diaries, direct/indirect calorimetry and accelerometry devices, heart rate monitors, and pedometers (18). Hence, there are two general approaches for measuring PA, namely, objectively and subjectively, which are distinguished on how the data are collected. Subjectively measured PA is mainly assessed using self-reported data whereas objectively measured PA data are regularly provided by devices such as accelerometers or heart rate monitors (19, 20). When determining PA in everyday life, accelerometers are suitable to precisely record the duration, frequency, and intensity of the activity (19). However, when we need to also measure the type and context of the activity, questionnaires can be used. Both measurement methods offer advantages and limitations. For example, various activities cannot be accurately recorded by the accelerometer (e.g., static activity or cycling) and questionnaires can be influenced by memory gaps or incorrect information owing to, e.g., social desirability (21). As objective and subjective PA show low to moderate correlation values (22), and there often is a discrepancy between accelerometer-based measured and one’s self-reported PA (20), it is of particular interest to include and compare the results from both measurement instruments in this study.

As recently published, the children and adolescents of Luxembourg are insufficient physically active and only 62 of the 242 students of a study fulfill the WHO guideline (23). However, in this study the data were not analyzed in terms of a potential seasonal effect and, more importantly, to our best knowledge, no studies are available, in which a seasonal comparison of both accelerometer-based and self-reported PA data has been conducted within one study. Nevertheless, based on questionnaires previous studies revealed lower self-reported physical activity in winter (24), and further studies revealed lower objective activity in the colder season measured by accelerometers (13).

The purpose of the present study was to assess the daily PA time of children and adolescents in Luxembourg during the autumn/winter season as well as during the spring/summer season, and to examine if there is a seasonal influence on PA behavior or level measured objectively by accelerometers and subjectively by questionnaires. Based on the results of previous studies mentioned previously, we hypothesize that children and adolescents of Luxembourg are less physically active in autumn/winter than in spring/summer. Another aim was to investigate whether the relation of accelerometer-based and self-reported levels of PA remain stable or if any changes occur throughout the year. Due to various studies finding self-reported or objectively measured lower PA in winter, and the knowledge that the self-reported PA score often shows higher values (18), we assume that there is no seasonal effect with regard to the relationship between the accelerometer-based and the self-reported PA.

## Methods

### Participants

Four primary and five secondary schools in Luxembourg took part in this study. The schools were randomly selected from different regions of Luxembourg to represent urban and rural areas. Overall, 24 classes from different grades (nine classes from Grade 4 and five classes each from Grades 7, 5, and 3) participated in the study. Due to the longitudinal design, one class of Grade 3 could not be included in the analyses because no student of this class provided valid data at both measurement times. Hence, data of 23 classes were included.

A total of *N* = 325 students between the ages of 10 and 18 years participated in the first wave (T1) of this longitudinal study including two measurement periods. At the second wave (T2), *N* = 21 (6.46%) participants dropped out (e.g., due to illness or change of school/classes). As attrition rates from 30% to 70% are often reported in longitudinal studies [e.g., (25)], a dropout rate of less than 10% can be considered negligible. Of the remaining *N* = 304 students, a total of *N* = 137 students [81 female students (59.12%); 56 male students (40.8%)] with an average age of *M =* 12.37 (*SD* = 2.16) years fulfilled the inclusion criteria at both measurements (T1 and T2), and were therefore included in the analysis. Inclusion criteria refer to, *inter alia*, wearing the accelerometer at least 8 h over 4 days including one weekend day (26) and completing the questionnaire.

### Measure

Personal data were collected by asking the participants about their gender and age personally. Height and weight were measured by trained personnel.

Accelerometer-based PA (MVPA per day and SED time per day) was measured using the accelerometer ActiGraph wGT3X-BT (ActiGraph LLC, Pensacola, FL, USA), a small and easy-to-wear device that measures the acceleration of the body in different spatial dimensions. In recent years, the ActiGraph accelerometer was the most frequently used in research and has shown good validity and reliability (27).

Self-reported PA was assessed using one item of the MoMo PA questionnaire (28). In this study, only the item for overall PA capturing information on numbers of days (1–7) of at least 60 min of PA during a typical week was considered [“In a typical week how many days are you physically active for at least 60 minutes (excluding physical education)?”]. One item with the wording “How many minutes per day are you physically active?” was added.

Weather data (temperature and rainfall) were taken via the weather and climate database https://meteostat.net/.

### Procedure

Each respective wave of data collection lasted for almost 2 months. The first data collection took place during the first trimester of the participants' school year in autumn/winter (T1: October to December 2018) and the second data collection, consisting of the same procedure with the accelerometer and the questionnaire, was performed six months later during the third trimester of the participants' school year in spring/summer (T2: May to July 2019).

The accelerometers were distributed by trained personnel to each individual participant during the school class. The students individually received detailed information about the accelerometer, and they were instructed to wear the accelerometer on the right hip for seven consecutive days while being awake, and to remove it only for water-based activities and while sleeping. Each accelerometer was previously initialized at a 30 HZ frequency. In addition, participants' height and weight were measured and stickers helping remember to wear the accelerometer every day were given to the participants. They were also given a protocol that explained how to record the times when they did not wear the accelerometer and their reasons, the time when they woke up, and the time when they went to sleep. After 1 week, the accelerometers were collected during a school lesson and potential ambiguities were clarified.

The selected item of the MoMo PA questionnaire and the additional item concerning the time of physical activity per day were completed digitally using the secured platform OASYS (29) from the University of Luxembourg. Due to the advantage of a digital testing, no missing data needs to be reported. The questionnaires were filled out at school during the same class when the distribution of the accelerometer took place and were supervised by trained personnel, who were available to answer questions or provide assistance.

All participants signed informed consent forms, while written permission was additionally obtained from the legal representatives of all participants younger than 16 years. The study was conducted in accordance with the Declaration of Helsinki and the European data protection directive and was approved by the Ethics Review Panel of the University of Luxembourg.

### Data processing and analyses

The accelerometer data were downloaded, processed, and analyzed after the 7-day period using the software ActiLife v6.13.4 (Actigraph Inc., USA). Based on the cut-off points given by Evenson et al. (30), the average time spent in MVPA, as an indicator of the PA behavior of children and adolescents, and being sedentary were calculated. Time spent not wearing the accelerometer was identified by the algorithm by Choi et al. (31).

The statistical tests were conducted using SPSS 21 (IBM, SPSS Inc., Chicago, IL, USA). Means, standard deviations, and frequencies were calculated to describe the data. Repeated measures ANOVA were computed to analyze differences in PA between T1 (autumn/winter) and T2 (spring/summer) with season as the main factor and gender as a covariate. Dependent variables were accelerometer-based PA (MVPA per day and SED time per day) and self-reported PA per day.

Unpaired *t-*tests were computed to analyze differences in self-reported and accelerometer-based PA, respectively, between male and female students for each season. Paired *t-*tests were conducted to analyze differences in self-reported PA between seasons. To determine the effect size, Cohen's d was calculated. Except for SED time per day, the variables were not normally distributed. However, both the repeated measures ANOVA and the *t*-test are considered to be very robust against the violation of the normality assumption and there was no further violation of the requirements (32).

Difference scores between self-reported and accelerometer-based PA were calculated to describe their relation, whereby the self-reported PA time, which corresponds to the accelerometer-based PA in a range of ±5 min, was rated as correct. Spearman correlation was calculated among all variables and specifically for the difference scores to analyze the relation between self-reported and accelerometer-based PA in terms of stability. The level of significance was set at *p* < 0.05.

## Results

### Participants

Participants’ age and anthropometric data are shown in Table 1. It has to be considered that at T1 *N* = 242 students and at T2 *N =* 137 students delivered valid data. The *N *= 105 students giving no valid data at T2 were significantly older than the *N* = 137 included in the analysis. Nevertheless, neither MVPA per day or SED time per day nor self-reported PA per day were significantly different at T1 between the students giving valid data at both measurements and the students not giving valid data at T1 and T2. Therefore, it can be assumed that both subsamples did not differ in terms of relevant variables.

**Table 1:** Anthropometric data.

Sample	Age (years)		Height (cm)		Weight (kg)	
	*M*	*SD*	*M*	*SD*	*M*	*SD*
Overall (*N* = 137)	12.78	2.22	159.03	12.79	53.58	18.29
Female students (*n* = 81)	13.09	2.34	160.28	10.67	56.25	18.61
Male students (*n* = 56)	12.34	1.97	157.47	14.94	49.71	17.26

Age at T1; height and weight as the average from T1 and T2.

### Accelerometer-based PA

On average, the daily time spent in MVPA was *M =* 49.80 (*SD* = 21.12) min at T1 (autumn/winter) and *M =* 53.81 (*SD* = 21.37) min at T2 (spring/summer; Table 2). At both measurements, the average MVPA is below the WHO guideline of at least 60 min/day. A repeated measures ANOVA revealed a significant seasonal effect on MVPA per day [*F*(1, 135) = 7.69, *p* < 0.05, partial *η*² = 0.054] and on gender [*F*(1, 135) = 25.10, *p* < 0.001, partial *η*² = 0.16].

**Table 2:** MVPA, self-reported PA, and SED time at T1 and T2.

Measurement period	Variable	Overall (*N* = 137)		Female students (*n* = 81)		Male students (*n* = 56)	
	* *	*M*	*SD*	*M*	*SD*	*M*	*SD*
T1 (autumn/winter)	MVPA/day (min)	49.80	21.12	43.09	17.44	59.50	22.33
	SED time/day (min)	585.02	92.95	596.78	73.96	568.02	113.61
	Self-reported PA/day (min)	74.61	56.78	69.27	58.73	82.32	53.42
T2 (spring/summer)	MVPA/day (min)	53.81	21.37	47.62	17.38	62.76	23.49
	SED time/day (min)	593.97	107.94	609.24	103.53	571.88	111.26
	Self-reported PA/day (min)	77.55	54.04	74.16	54.95	82.45	52.80

A significant gender difference was found at T1 [*t*(135) = 4.82, *p* < 0.001; *d* = 0.77] such that male students (*M* = 59.50, *SD* = 22.33 min) spent more time in MVPA per day than female students (*M* = 43.09, *SD* = 17.44 min) at T1. Likewise, male students accrued significantly [*t*(135) = 4.36, *p* < 0.001; *d* = 0.71] more MVPA minutes per day (*M* = 62.76; *SD* = 23.49 min) than female students (*M* = 47.62; *SD* = 17.38 min) at T2. However, there was no significant correspondence between season and gender [*F*(1, 135) = 0.204, *p* = 0.652].

The average SED time per day was *M* = 585.2 (*SD* = 92.95) min at T1 and *M* = 593.97 (*SD* = 107.94) min at T2. No significant seasonal effect [*F*(1, 135) = 0.91, *p* = 0.340] could be revealed. However, a significant effect for gender could be found [*F*(1, 135) = 4.79, *p* < 0.05, partial *η*² = 0.03].

A significant gender difference was found only at T2 [*t*(135) = −2.01, *p* < 0.05; *d* = 0.34], showing that female students accrued more SED time (*M =* 609.24, *SD =* 103.53 min) than male students (*M =* 571.88, *SD =* 111.26 min). There was no significant difference [*t*(135) = −1.79, *p* = 0.075] between the SED time of the male students (*M =* 568.02, *SD =* 113.61 min) and the female students (*M =* 596.78, *SD =* 73.96 min) at T1. No correspondence between season and gender could be revealed [*F*(1, 135) = 0.25, *p* = 0.614].

### Self-reported physical activity

**Figure 1:**
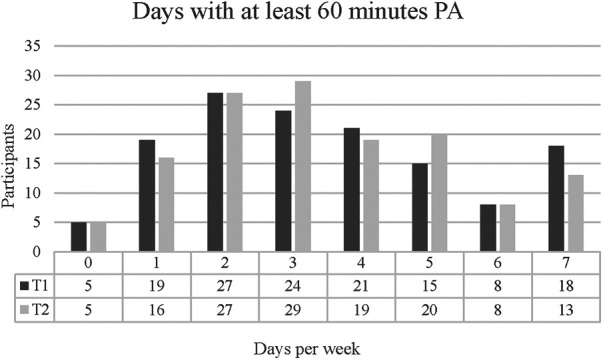
Self-reported days with at least 60 min PA at T1 and T2 during a typical week.

Overall, 27 (19.7%) students reported to be typically active on two days per week at T1 and T2. A total of 24 (17.5%) students reported being active on three days per week at T1, and 29 (21.2%) at T2. Five students (3.6%) reported to be never active at T1 and at T2 at all, and 18 (13.1%) reported to be active every day at T1 and 13 (9.5%), at T2. The exact distribution of the specified days with PA is shown in Figure 1. A paired *t*-test revealed no difference between T1 (*M =* 3.49, *SD =* 2.02 days) and T2 [*M =* 3.45, *SD =* 1.89 days; *t*(136) = .288, *p* = .773].

On average, *M =* 74.61 (*SD =* 56.78) min of PA per day for T1 and *M =* 77.55 (*SD =* 54.04) min for T2 were reported (Table 2). A repeated measures ANOVA revealed no seasonal effect on self-reported PA per day [*F*(1, 135) = 0.21, *p* = .646]. Female students reported being active *M =* 69.27 (*SD =* 58.73) min at T1 and *M =* 74.16 (*SD =* 54.95) min at T2, whereas male students indicated higher values with *M =* 82.32 (*SD =* 53.42) min at T1 and *M =* 82.45 (*SD =* 52.80) min at T2. However, no significant gender differences were found, neither for T1 [*t*(135) = 1.326, *p* = .187], nor for T2 [*t*(135) = .882, *p* = .380]. Likewise, no correspondence effect between season and gender could be determined [*F*(1, 135) = 0.19, *p* = .662].

### Relation between accelerometer-based and self-reported physical activity

In terms of the relation between accelerometer-based and self-reported daily PA time, at T1 11.7% of the students reported a daily PA time that complies within the ±5 min range with the accelerometer-based MVPA. Compared with the accelerometer-based MVPA, 60.6% reported more PA and 27.7% reported less PA. At T2, the values changed slightly to 15.3% showing compliance, 55.5% reporting more PA, and 29.2% reporting less PA. The majority of 62.7%, who reported more PA than the accelerometer-based PA at T1 maintained their higher self-reported PA levels at T2. However, 15.7% of this group reported a PA time that corresponds to their accelerometer-based PA and 21.7% even reported less PA at T2.

The mean difference scores between the accelerometer-based and the self-reported PA at T1 (*M =* 24.81, *SD =* 54.09 min) and T2 (*M =* 23.74, *SD =* 53.00 min) were significantly correlated (*r* = .31, *p *< .001) indicating a relatively stable relationship between both PA measures over time. However, this correlation is mainly due to the female students. Their mean difference scores at T1 (*M =* 26.18, *SD =* 56.75 min) and T2 (*M =* 26.54, *SD =* 52.33 min) were strongly correlated (*r* = .51, *p *< .001), while the difference scores of the male students at T1 (*M =* 22.82, *SD =* 50.46 min) and T2 (*M =* 19.69, *SD =* 54.17 min) show no correlation (*r* = .02, *p *= .891).

The correlation coefficients among all measures are presented in Table 3.

**Table 3:** Intercorrelations of the study variables.

	1	2	3	4	5	6	7	8	9	10
1. MVPA/day (T1)	—									
2. SED time/day (T1)	−**0.53**	—								
3. Self-reported PA/day (T1)	**0**.**31**	−0.14	—							
4. Self-reported days with PA (T1)	**0**.**34**	−**0**.**30**	**0**.**37**	—						
5. MVPA/day (T2)	**0**.**72**	−**0**.**48**	**0**.**27**	**0**.**38**	—					
6. SED time/day (T2)	−**0**.**37**	**0**.**64**	−0.07	−**0**.**30**	−**0**.**38**	—				
7. Self-reported PA/day (T2)	**0**.**22**	−**0**.**21**	**0**.**39**	**0**.**25**	**0**.**25**	−0.11	—			
8. Self-reported days with PA (T2)	**0**.**38**	−**0**.**27**	**0**.**36**	**0**.**61**	**0**.**42**	−**0**.**24**	**0**.**41**	—		
9. Difference between MVPA and self-report (T1)	−0.16	0.06	**0**.**85**	**0**.**19**	−0.07	0.08	**0**.**27**	0.14	—	
10. Difference between MVPA and self-report (T2)	−0.14	0.01	**0**.**26**	0.07	−**0**.**23**	0.08	**0**.**85**	0.16	**0**.**31**	‒

Bold values indicate statistically significant value (*p* < 0.05).

### Weather

At T1, the average temperature was 7.8° Celsius (*SD =* 5.2° Celsius) with an absolute minimum at −3.5° Celsius and an absolute maximum at 24.5° Celsius. The total amount of precipitation was 177.6 mm with an average of 2.3 mm (*SD =* 6.17 mm). At T2, the average temperature was higher (*MW =* 15.3°C, *SD =* 5.6°C) with an absolute minimum at 0.1°C and an absolute maximum at 34.7°C. The total amount of precipitation was lower (143.2 mm) with an average of 1.9 mm (*SD =* 4.43 mm).

## Discussion

The purpose of this study was to assess the daily PA of children and adolescents in Luxembourg during the autumn/winter and the spring/summer seasons and to examine if there is a seasonal influence on the PA behavior. A seasonal comparison of PA in Luxembourg has never been conducted before, and thus, it was important to collect accelerometer-based data of both autumn/winter and spring/summer, for the first time. Moreover, and to our best knowledge, there are no studies available in which a seasonal comparison of both the accelerometer-based and the self-reported PA has been conducted.

According to previous investigations in Luxembourg (5, 23, 33), the PA of children and adolescents is not sufficient when compared with the WHO recommendation. As we found that the average time spent in MVPA was *M =* 49.80 (*SD =* 21.12) min/day in autumn/winter and *M =* 53.81 (*SD =* 21.37) min in spring/summer, the previous results could be confirmed for both seasons. We detected a significant difference in MVPA between autumn/winter and spring/summer, which discloses a decreased PA of the children and adolescents of Luxembourg in autumn/winter. This result is similar to previous studies revealing a seasonal difference in PA with lower PA in winter or colder seasons (34–36). Since previous studies revealed that periods of infrequent participation in PA are linked to becoming physical inactive (37), this is a crucial finding when it comes to maintaining or promoting PA among children and adolescents, especially when considering the already insufficient level of PA.

In contrast to MVPA, we found no seasonal variation in SED time. On average, the children and adolescents of Luxembourg spent *M = *585.02 (*SD =* 92.95) min in autumn/winter and *M = *593.97 (*SD =* 107.94) min in spring/summer being sedentary, which is contrary to other studies revealing a higher SED time in winter [e.g., (34)]. Our results with no change in SED time per day indicate that there is probably no change in overall behavior but rather sport or PA on the MVPA level varies seasonally. One reason for this could be that Luxembourg has a milder climate in autumn/winter than, for example, in other countries such as Denmark with an increased SED time in winter (34). Hence, it has to be taken into account, that the association between season and PA and SED time is likely to be country-specific, because the weather varies widely between countries (17, 38).

Regarding the weather, there are a few studies in which participants mentioned that bad weather was a barrier to PA, and in some studies, only a few individuals have indicated that weather impedes PA (38). However, further investigations should focus on the detailed reasons for less PA in autumn/winter.

Another aim was to evaluate the self-reported PA behavior in autumn/winter and spring/summer and examine whether the relation of the accelerometer-based and the self-reported PA remains stable over seasons. In contrast to other studies [e.g., Bélanger et al. (24)], we found no significant difference in self-reported PA between seasons. In this study, neither the self-reported PA per day nor the reported days with physical activity showed significant differences in this study.

In autumn/winter, only 11.7% of the children and adolescents reported a daily PA that complies with the accelerometer-based PA and in spring/summer, 15.3% stated a PA that corresponds to their accelerometer-based PA. This is in line with studies showing that most people tend to report more PA than their accelerometer-based PA reveals (18). In this analysis, the mean difference scores between the accelerometer-based and the self-reported PA in autumn/winter (*M =* 24.81, *SD =* 54.09 min) and in spring/summer (*M =* 23.74, *SD =* 53.00 min) correlated between the seasons. Thus, these results show that the discrepancy between the accelerometer-based and the self-reported PA is a relatively time-stable phenomenon. Although the difference remains relatively stable throughout the analyzed seasons, it is interesting that the children and adolescents do not perceive their PA as less in the colder season as in the warmer season. This result appears to be crucial information for future interventions, because children and adolescents are apparently not aware of the objectively measured lower level of PA in the colder season and they should be enlightened first.

However, it must be considered, that 60.6% (T1) and 55.5% (T2) of the children and adolescents reported more PA than the accelerometer-based PA and this shows that throughout the whole year self-reported PA is higher than the accelerometer-based PA. The tendency toward reporting more PA than the accelerometer-based PA score has already been shown in other studies (18, 21).

One limitation of this study is that some sports (e.g., swimming) could not be involved in the analyses because the accelerometer could not be worn during this kind of exercise. Furthermore, although ActiGraph accelerometers have been shown to validly measure the PA of children and adolescents (39), there are some activities that are not well detected (e.g., static exercises), and hence, the children's actual PA levels could be underestimated. However, this would affect autumn/winter and spring/summer equally, and therefore, can be neglected for the seasonal analyses of the accelerometer-based PA. Another aspect to be considered is the impact of seasons and explicit weather conditions. No exact metrological data were recorded and directly included in the analysis. As T1 took place from October to December, it is neither clearly autumn nor clearly winter and the same applies to T2, which is unclear whether it is spring or summer. Nevertheless, previous research shows that PA is lower in autumn and winter than in spring and summer (14). Hence, this first study of seasonal PA data in Luxembourg enables an analysis between colder and warmer seasons. It also has to be considered that there might be differences in seasonal variations of PA between urban and rural children and adolescents (40). However, as schools were randomly selected from five regions of Luxembourg, the participants should represent the students of Luxembourg from urban and rural areas equally.

## Conclusion

With this first seasonal comparison of PA in Luxembourg, we detected that children and adolescents are less physically active in autumn/winter than in spring/summer. Therefore, schools, sports clubs, and communities should offer special PA programs for this season, which are independent from climatic conditions and equally suitable for females and males. Providing indoor opportunities during the colder season, for example, may foster regular PA. Therefore, municipalities could provide sports halls for free use for children and adolescents or gyms could offer special rates for the colder season.

Measuring PA throughout the year enables monitoring the activity behavior more accurately and may help further developing such programs. The phenomenon of the discrepancy between the accelerometer-based and the self-reported PA being relatively time-stable is a crucial aspect in the practice of PA and health promotion and rehabilitation.

## Data Availability

The raw data supporting the conclusions of this article will be made available by the authors, without undue reservation.
